# Efficacy and Safety of Transcranial Magnetic Stimulation for Attention‐Deficit Hyperactivity Disorder: A Systematic Review and Meta‐Analysis

**DOI:** 10.1002/brb3.70246

**Published:** 2025-01-19

**Authors:** Binbin Fu, Xiangyue Zhou, Xuan Zhou, Xin Li, Zhengquan Chen, Yanbin Zhang, Qing Du

**Affiliations:** ^1^ Department of Rehabilitation, Xinhua Hospital Shanghai Jiao Tong University School of Medicine Shanghai China; ^2^ Chongming Branch of Xinhua Hospital, School of Medicine Shanghai Jiao Tong University Shanghai China; ^3^ Institute of Rehabilitation Engineering and Technology University of Shanghai for Science and Technology Shanghai China

**Keywords:** attention‐deficit hyperactivity disorder, hyperactivity, impulsivity, inattention, transcranial magnetic stimulation

## Abstract

**Background:**

Transcranial magnetic stimulation (TMS) is a promising neuromodulation technique that has been widely used in neuropsychiatric disorders, but there was no evidence on its effect on the improvement attention‐deficit hyperactivity disorder (ADHD).

**Objective:**

This systematic review and meta‐analysis aimed to investigate the efficacy and safety of TMS in reducing ADHD symptoms.

**Method:**

We systematically searched four databases (PubMed, Embase, Web of Science, and Cochrane Library databases) for randomized controlled/crossover trials on the efficacy and safety of TMS on ADHD symptom improvement compared to sham rTMS or non‐TMS interventions, published until September 18, 2024. Extracted data from the included studies involved patient characteristics, intervention protocols, and main outcomes. The effect size of the TMS treatment was evaluated using the standardized mean difference (SMD) with a 95% confidence interval (CI), calculated with either a random effects model or fixed effects model depending on the level of heterogeneity.

**Result:**

Eight studies (325 ADHD patients in total) were included in this systematic review and meta‐analysis. According to the core symptoms, TMS significantly improved inattention (SMD = −0.94, 95% CI = −1.33 to −0.56, *p* < 0.001) and hyperactivity/impulsivity (SMD = −0.98, 95% CI = −1.27 to −0.69, *p* < 0.001) compared to non‐TMS interventions after 3–6 weeks of intervention. During the 1‐month follow‐up, the TMS group still demonstrated a significant improvement in inattention symptoms compared to the non‐TMS group (SMD = −0.67, 95% CI = −1.06 to 0.28, *p* < 0.001). The total symptoms in the TMS group only showed improvement in the 1‐month follow‐up compared to the non‐TMS group. (SMD = −0.48, 95% CI = −0.82 to −0.14, *p* = 0.005). Only minor adverse events were reported in the included studies, comprising headache and scalp discomfort.

**Conclusion:**

TMS significantly improved the inattention, hyperactivity/impulsivity, and total symptom scores in ADHD patients with minor adverse events. Future research should focus on the association between different brain regions and symptoms in ADHD patients, which is crucial for stimulation navigation in TMS interventions. The trial is registered in PROSPERO (PROSPERO registry number: CRD42023473853).

## Graphical abstract

This systematic review and meta‐analysis found that transcranial magnetic stimulation (TMS) significantly improved the symptoms of attention‐deficit hyperactivity disorder (ADHD), including inattention, hyperactivity/impulsivity, and total symptoms, with minimal adverse events.

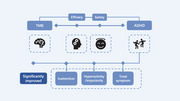

## 1 Introduction

Attention‐deficit hyperactivity disorder (ADHD) is one of the most common neuropsychiatric disorders in children (Sayal et al. 2018; van Rooij et al. 2015), occurring at any stage of life. Globally, the prevalence of ADHD in children, adolescents, and adults is approximately 7.6%, 5.6% (Salari et al. 2023), and 2.58% (Song et al. 2021), respectively. According to the fourth edition of the *Diagnostic and Statistical Manual of Mental Disorders* (DSM‐IV), the core symptoms of ADHD are pervasive and frequent inattention and hyperactivity/impulsivity (American Psychiatric Association [APA] 2013). These symptoms define three distinct presentations of the disorder: predominantly inattentive, predominantly hyperactive‐impulsive, and a combined presentation. Research reveals that predominantly inattentive presentations (ADHD‐I) and combined (ADHD‐C) are the ones most commonly encountered (Vitola et al. 2017; Willcutt 2012). And nearly 60% of patients diagnosed in childhood still present symptoms in adulthood (Adler et al. 2017; Kessler et al. 2010). Beginning in childhood, ADHD can seriously impact study and work, and the behavioral problems caused by ADHD in childhood predict varying degrees of impairment of academic and social functioning throughout childhood and adolescence (Agnew‐Blais et al. 2021, 2016), including poor academic performance, substance use disorders, delinquency, and even suicide (Fitzgerald et al. 2019; Fleming et al. 2017; Groenman, Janssen, and Oosterlaan 2017; Mohr‐Jensen et al. 2019; Ros and Graziano 2018).

ADHD patients exhibited broad impairments in right and left hemispheric dorsals (Chambers, Garavan, and Bellgrove 2009; Corbetta, Patel, and Shulman 2008), ventral (Sripada, Kessler, and Angstadt 2014), and medial fronto‐cingulo‐striato‐thalamic (Chantiluke et al. 2014; Rubia, Alegria, and Brinson 2014), as well as fronto‐parieto‐cerebellar networks (van Rooij et al. 2015). These networks are involved in cognitive control, attention, timing, and working memory, demonstrating cognitive‐domain dissociated patterns. The first‐line treatment for ADHD includes pharmacotherapy. Central nervous system (CNS) stimulants and non‐CNS stimulants have been used to treat ADHD (Elliott et al. 2020; Golmirzaei et al. 2016; Iwanami et al. 2020). CNS stimulants block the reuptake of dopamine and norepinephrine neurotransmitters by the presynaptic membrane (Golmirzaei et al. 2016), which enhances the function of the striatum and the prefrontal cortex (PFC) (Faltinsen et al. 2018), and non‐CNS stimulants can also act centrally to enhance neurotransmitter concentrations (Elliott et al. 2020; Iwanami et al. 2020). However, about 20% of ADHD patients may experience insufficient efficacy or be intolerant to side effects such as sleep disturbances, nausea, xerostomia, headache, and irritability (Pozzi et al. 2020).

Noninvasive brain stimulation technology including repetitive transcranial magnetic stimulation (rTMS) and transcranial direct current stimulation (tDCS) can be used for neuropsychological diseases, mainly focusing on the regulation of central neurotransmitters and the remodeling of the center (He et al. 2020). Compared to rTMS, tDCS is more commonly used in neurodevelopmental disorders, especially ADHD (Leffa et al. 2022; Salehinejad et al. 2020). The underlying neural mechanisms of ADHD involve the cingulo‐frontal‐parietal (CFP) brain network, which includes the dorsal‐anterior mid‐cingulate cortex (daMCC), dorsal lateral prefrontal cortex (DLPFC), ventrolateral prefrontal cortex (VLPFC) and parietal cortex (Bush 2011). Some studies found that PFC played a crucial role in cognitive function in ADHD patients (Alyagon et al. 2020; Viering et al. 2022). The importance of PFC in the neuropathological mechanisms of ADHD suggests that electrical/magnetic stimulation of PFC may contribute to the recovery of core symptoms of ADHD (L. Chen et al. 2016; Hart et al. 2013; Lukito et al. 2020; Norman et al. 2016; Rubia 2018; Wasserstein and Stefanatos 2016). Also the hypoactivity of the right inferior frontal gyrus (IFG) has been linked to impaired executive functions observed in individuals with ADHD (Breitling‐Ziegler et al. 2021). Various evidence suggest that the regulatory effects of NIBS may be mediated through mechanisms of plasticity (He et al. 2020). rTMS and tDCS have been demonstrated to evoke long‐term potentiation (LTP) or long‐term depression (LTD) in the stimulated areas of the brain (de Boer et al. 2021; Huang et al. 2017). This can be verified by following up on the sustained efficacy in ADHD patients.

As a promising neuromodulation technique, rTMS offers an adjunctive treatment option for patients with ADHD. However, relative to tDCS, the literature on rTMS for the treatment of ADHD is sparse and lacks updating, especially with regard to comparisons with medications or other means of treating ADHD. There is no clear consensus on whether rTMS improves ADHD symptoms and is superior or safer than other treatments. Therefore, the aim of this systematic review and meta‐analysis is to investigate the efficacy and safety of rTMS on inattention, hyperactivity/impulsivity, and total ADHD symptoms in patients with ADHD compared to sham rTMS or non‐TMS means. Our hypothesis is that rTMS may significantly improve ADHD symptoms when compared to sham rTMS or non‐TMS treatment.

## 2 Methods

This systematic review and meta‐analysis adhered to the guidelines of the Preferred Reporting Items for Systematic Reviews and Meta‐Analysis (PRISMA) and the Cochrane Collaboration's recommendations. The study protocol has been officially documented in the PROSPERO database (Prospero registry number: CRD42023473853).

### 2.1 Retrieval Strategy and Selection Process

We searched for PubMed, Embase, Web of Science, and Cochrane Library databases until September 18, 2024, without restrictions on country, or publication years. The keywords used are “transcranial magnetic stimulation,” “TMS,” “attention‐deficit hyperactivity disorder,” and “ADHD,” to identify studies concerning the efficacy and safety of TMS on ADHD. The complete search terms can be found in Table S1. In addition, we supplemented the electronic search by manually reviewing reference lists of the included studies to identify potentially eligible studies.

Endnote software version 20.0 (Clarivate, Philadelphia, PA, USA) was utilized for managing literature. After removing duplicate literature, two reviewers (B.F. and X.Z.) independently conducted an initial screening of pertinent studies based on the title and abstract, after which the remaining studies were subjected to full‐text examination. The discrepancies between the two reviewers were resolved through discussion, and if necessary, a third reviewer (Z.C.) would be consulted for arbitration.

### 2.2 Eligibility Criteria

Using the population, intervention, comparison, outcomes, and study design (PICOS) framework, the eligibility criteria were as follows:

(1) Populations: Children, adolescents, or adults who have been clinically diagnosed with ADHD by a specialist, using the *Diagnostic and Statistical Manual of Mental Disorders* (DSM) (APA 2013; Guze 1995). Participants with contraindications to TMS, such as epilepsy, brain trauma, or brain metal implants would be excluded. (2) Interventions: Studies utilizing TMS either as monotherapy or in combination with others and provided a clearly defined protocol containing information on the type, device, coil, location, intensity, frequency, total pluses, sessions, time, and duration. (3) Comparisons: sham‐rTMS or non‐TMS control conditions, including waitlist, medications or education. (4) Outcomes: The primary outcomes were the ADHD inattention symptom and hyperactivity/impulsivity symptom. The secondary outcome comprised total symptom scores. The severity of ADHD symptoms mentioned above can be reflected by the scores of the questionnaire. Eligible questionnaires included the DSM‐IV ADHD questionnaire, the Conners' Adult ADHD Rating Scale (CAARS), and the Swanson, Nolan, and Pelham, Version IV (SNAP‐IV). The frequency and severity of side effects were also collected. (5) Study: randomized controlled trials or randomized crossover trials that were written in the English language.

### 2.3 Data Extraction

The data extracted from the included studies encompassed author, years, country, setting, study design, patient characteristics (diagnosis criteria, interventions, age, sample size, gender, dropout rate), intervention protocol (TMS, sham rTMS, and non‐TMS treatment protocols), and outcome measures. For crossover studies, data from the initial part of the trial (precrossover) were favored.

Two authors independently executed data extraction. If the opinions were inconsistent, a third reviewer would reevaluate the articles and discuss them with the two reviewers to reach an agreement. The mean, standard deviation (SD), and sample size were extracted for the outcome measures in each group (i.e., active and sham) for the pooled analysis. In the absence of data, the researchers contacted the authors.

### 2.4 Risk of Bias Assessment

The risk of bias (ROB) assessment for randomized controlled trials used the Cochrane ROB tool to assess parallel trials and cross‐over trials. All studies were classified as “low risk,” “high risk,” or “unclear risk” based on five key domains of selection bias, performance bias, detection bias, attrition bias, and other biases. Two authors independently assessed the ROB, and any discrepancies being resolved by consensus. We assessed the quality of the evidence using the Graded Assessment, Development and Evaluation of Recommendations (GRADE) system, which classifies the level of evidence as very low, low, moderate, or high (Schünemann et al. 2019).

### 2.5 Data Synthesis and Statistical Analysis

RevMan 5.4 software (Copenhagen: The Nordic Cochrane Center, The Cochrane Collaboration, 2014) was used for statistical analysis. The standardized mean difference (SMD) and 95% CI were used as the effect size indicators. The *χ* ^2^ test was used to determine the heterogeneity between the included studies. *p* ≥ 0.1 and *I* ^2^ < 50% suggested that small statistical heterogeneity and a fixed‐effect model would be used, while *p* < 0.1 and *I* ^2^ ≥ 50% suggested large heterogeneity with a random‐effects model being used for analysis. A *Z*‐test was used to assess the significance of the pooled effect.

## 3 Result

### 3.1 Search Results

**FIGURE 1:**
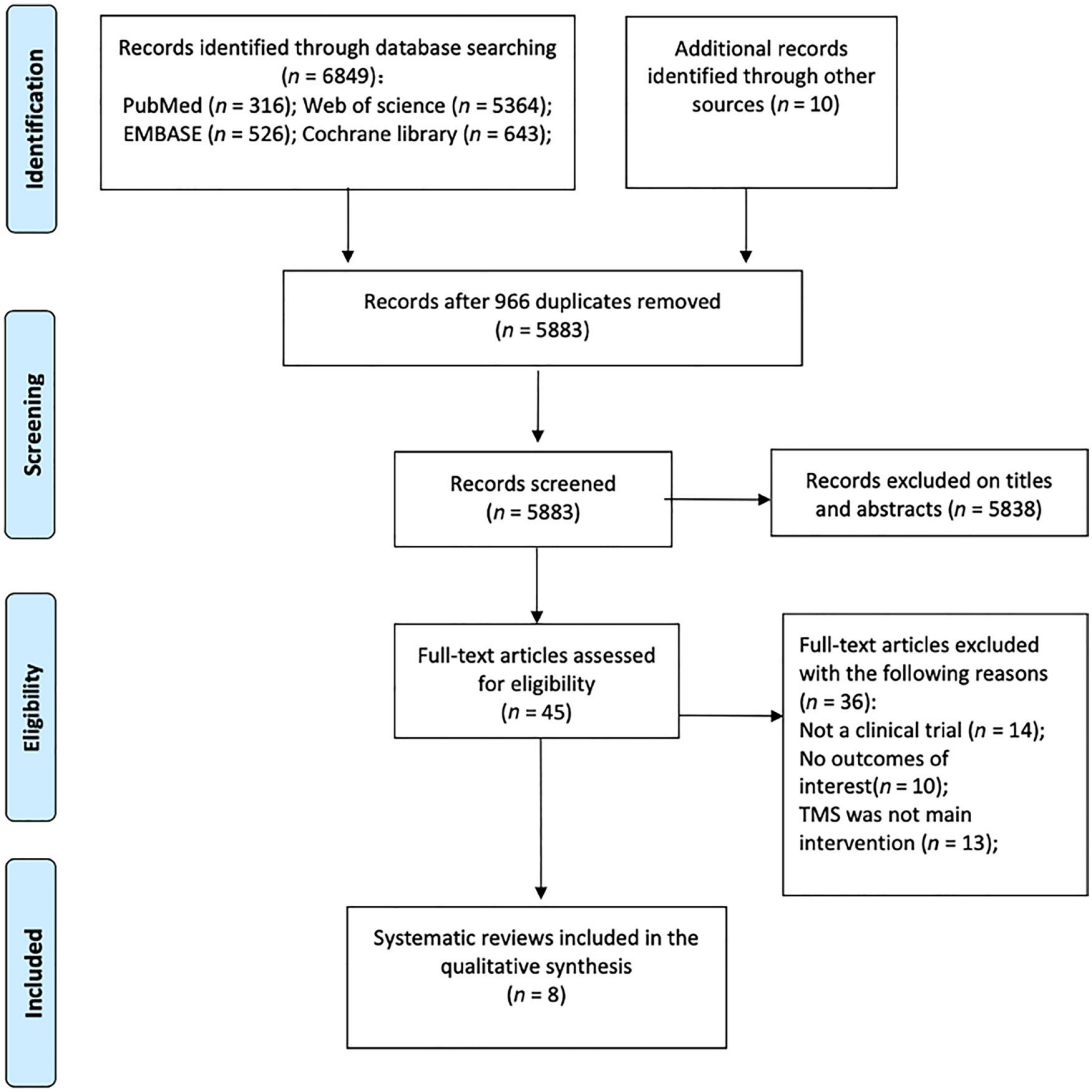
Flow diagram of the selection process.

The preliminary search yielded 6849 records, with 316 from PubMed, 5364 from Web of Science, 526 from EMBASE, and 643 from the Cochrane Library. And 10 were from additional records identified through other sources. Among them, 966 duplicate results were identified. In addition, a total of 5883 studies were excluded after the title/abstract screening. Among the remaining 45 articles, 36 were excluded after full‐text review (not a clinical trial: *n* = 14; no outcomes of interest: *n* = 10; TMS was not the main intervention: *n* = 13). Eight studies were included in the systematic review. The selection process is depicted in Figure 1.

### 3.2 Study Characteristics

Table 1 describes the characteristics of the population included in the study. Table 2 includes details for TMS and other interventions; Table 3 shows the main results of the included studies. Four studies included adults (Alyagon et al. 2020; Bleich‐Cohen et al. 2021; Bloch et al. 2010; Paz et al. 2018), and three studies included children (Cao et al. 2019, 2018, 2019, 2018; Nagy et al. 2022), with the other study recruiting both adults and children (Weaver et al. 2012) participants. Six studies used rTMS (Alyagon et al. 2020; Bloch et al. 2010; Cao et al. 2019, 2018, 2019, 2018; Kessler et al. 2010; Nagy et al. 2022; Weaver et al. 2012), and two studies used deep transcranial magnetic stimulation (dTMS) (Bleich‐Cohen et al. 2021; Paz et al. 2018), a form of rTMS that utilizes specially designed coils to depolarize neurons at a deeper level. PFC was included in the TMS stimulation site in all studies, and high‐frequency TMS (> 1 Hz) (Burke et al. 2019) was used in participants in all studies. The duration of the intervention ranged from 3 to 6 weeks. Regarding the treatment plans for the control group, all studies except for Cao et al. (2019, 2018, 2019, 2018) allowed ADHD medication during intervention. None of the studies reported to have included only medication‐naive patients. Atomoxetine (ATX) was used as a drug in three studies (Cao et al. 2019, 2018, 2019, 2018; Nagy et al. 2022), and the use of ATX was consistent, specifically, starting at a dose of 0.5 mg/kg/day, titrating to 1.2 mg/kg/day after 3 days, and maintaining this dose until the end of the intervention.

**TABLE 1:** Characteristics of the population included in the study.

Author, year	Country	Setting	Study design	Population	Diagnostic criteria	Interventions	Age (years)	Sample size [female]	Dropout rate
Nagy et al. (2022)	Egypt	The Institute of Psychiatry Ain Shams University	Double‐blind, sham‐controlled RCT	ADHD (children)	DSM‐IV	G1: TMS + ATX G2: sham + ATX	G1: 8.7 ± 1.76 G2: 8.47 ± 1.7	G1: *n* = 30 [8] G2: *n* = 30 [6]	14.29%
Bleich‐Cohen et al. (2021)	Israel	The Tel Aviv Sourasky Medical Center	Double‐blind, sham‐controlled RCT	ADHD (adults)	DSM‐V	G1: rPFC TMS G2: lPFC TMS G3: sham	G1: 35.6 ± 8.7 G2: 35.1 ± 10.1 G3: 34.7 ± 9.2	G1: *n* = 24 [7] G2: *n* = 22 [7] G3: *n* = 16 [8]	18.42%
Alyagon et al. (2020)	Israel	The Soroka University Medical Center	Semi‐blind, sham‐controlled RCT	ADHD (adults)	DSM‐V	G1: TMS G2: AC G3: sham	G1: 26.62±0.66 G2: 26.13±0.59 G3: 27.64±1.58	G1: *n* = 15 [13] G2: *n* = 14[10] G3: *n* = 14 [11]	17.31%
Cao et al. (2019)	China	The People's Liberation Army No. 102 Hospital and the Changzhou No. 2 People's Hospital	Double‐blind, sham‐controlled RCT	ADHD (children)	DSM‐V	G1‐1: TMS G1‐2: sham G2‐1: ATX G2‐2: placebo	ADHD: 8.83 ± 2.53 HC: 9.17 ± 2.26	G1‐1: *n* = 18 G1‐2: *n* = 16 G2‐1: *n* = 16 G2‐1: *n* = 16 HC: *n* = 18 [6]	12.00%
Cao et al. (2018)	China	Psychological Centre for Adolescents and Children at 102th Hospital of People's Liberation Army of China	Double‐blind, sham‐controlled RCT	ADHD (children)	DSM‐V	G1: TMS G2: ATX G3: TMS + ATX	G1: 8.36 ± 2.46 G2: 9.22 ± 2.39 G3: 8.50 ± 2.20	G1: *n* = 20 [2] G2: *n* = 19 [3] G3: *n* = 21 [6]	6.25%
Paz et al. (2018)	Israel	Shalvata Mental Health Care Center	Double‐blind, sham‐controlled RCT	ADHD (adults)	DSM‐V	G1: TMS G2: sham	G1: 32.11 ± 6.47 G2: 30.85 ± 6.82	G1: *n* = 9 [3] G2: *n* = 13 [5]	15.38%
Weaver et al. (2012)	America	Department of Child and Adolescent Psychiatry, The Children's Hospital of Philadelphia, 2nd Floor	Sham‐controlled, crossover RCT	ADHD (adults, children)	DSM‐IV	Phase1: TMS‐sham Phase2: Sham‐TMS	18.11 ± 1.88	*n* = 9 [3]	NA
Bloch et al. (2010)	Israel	Tel Aviv University and Shalvata Mental Health Center	Double‐blind, sham‐controlled, crossover RCT	ADHD (adults)	DSM‐IV	Visit1: TMS‐sham Visiti2: Sham‐TMS	> 18	*n* = 13 [6]	NA

Abbreviations: AC, active control; ADHD, attention‐deficit hyperactivity disorder; ATX, atomoxetine; DSM‐IV, the fourth edition of the *Diagnostic and Statistical Manual of Mental Disorders*; DSM‐V, the fifth edition of the *Diagnostic and Statistical Manual of Mental Disorders*; G, group; HC, healthy control; lPFC, left prefrontal cortex; NA, not available; RCT, randomized controlled trials; rPFC, right prefrontal cortex; TMS, transcranial magnetic stimulation.

**TABLE 2:** Details for TMS and other interventions.

Author, year	Details for TMS										Details for interventions other than real TMS group
	Type	Device	Coil	Location	Intensity (% of MT)	Frequency (Hz)	Total pluses	Sessions	Time	Duration	
Nagy et al. (2022)	rTMS	Magventure R 30 stimulator	Figure‐8 coil	rDLPFC	90	10	2000	15	4 s on‐train, 26 s off inter‐train interval	3W	Sham TMS: same coil and location without touching the scalp ATX: 1.2 mg/kg·day
Bleich‐Cohen et al. (2021)	dTMS	Brainsway, IL	H6 coil	G1. rPFC G2. lPFC	120	18	1440	15	2 s per train, 20 s inter‐train interval	3W	Sham TMS: same coil and location without magnetic pulses
Alyagon et al. (2020)	rTMS	Magstim rapid^2^ stimulator	G1: H6 coil G2: Figure‐8 coil	rPFC (VLPFC /DLPFC)	120	18	1440	15	2 s long, 20 s apart	3W	AC stimulation: Use the separated coil Sham TMS: Use a sham coil to induce auditory artifact but a non‐penetrating electromagnetic field
Cao et al. (2019)	rTMS	Magneuro100 magnetic stimulator	Figure‐8 coil	rDLPFC	100	10	2400	30	4 s stimulation time, 26 s interval	6W	Sham TMS: Same simulation parameters and the coil was simply placed perpendicular to the scalp of the stimulation site ATX: 1.2 mg/kg·day
Cao et al. (2018)	rTMS	Magneuro100 magnetic stimulator	Figure‐8 coil	rDLPFC	100	10	2000	30	4 s stimulation, 26 s interval	6W	ATX:1.2 mg/kg·day TMS + ATX: same rTMS stimulation parameters and the same ATX dose
Paz et al. (2018)	dTMS	Brainsway, IL	H5 coil	PFC	120	18	1980	20	2 s per train, 20 s inter‐train interval	4W	NA
Weaver et al. (2012)	rTMS	Magstim rapid stimulator (Whitland, UK)	Figure‐8 coil	rDLPFC	100	10	2000	10	4 s on‐train, 26 s off intertrain interval	4W	Sham TMS: The figure‐8 coil was tilted at 90°
Bloch et al. (2010)	rTMS	A magstim super rapid stimulator	Figure‐8 coil	rDLPFC	100	20	NA	NA	2 s stimulation, 30 s inter‐stimulus interval	NA	Sham TMS: the same stimulation parameters with one wing of the figure‐8 coil in contact with the scalp and at a 45° angle with respect to the head

Abbreviations: AC, active control; ATX, atomoxetine; DLPFC, dorsolateral prefrontal cortices; dTMS, deep transcranial magnetic stimulation; lPFC, left prefrontal cortex; MT, motor threshold; NA, not available; PFC, prefrontal cortex; rDLPFC, right dorsolateral prefrontal cortices; rPFC, right prefrontal cortex; rTMS, repetitive transcranial magnetic stimulation; S, second; TMS, transcranial magnetic stimulation; VLPFC, ventrolateral prefrontal cortices; W, week.

**TABLE 3:** Outcome measures and key findings of the included studies.

Author, year	Assessment time points	Outcome indicators	Key findings	Safety findings
Nagy et al. (2022)	Pre, post, FU‐1M	CPRS‐R‐L, CGAS, CGI	The rTMS group showed greater improvement than the sham group in inattention, total ADHD severity, CGI, and CGAS after rTMS	NA
Bleich‐Cohen et al. (2021)	Pre, post, FU‐1M, FU‐2M	Clinical assessment: CAARS, CGI, AAQoL, BDI, BRIEF‐A, mindstreams Neural effects: WM and resting‐state paradigms	The rPFC dTMS group showed larger symptom improvement in the CAARS (self‐report) inattention/memory sub‐scale, as well as increased activations in the rDLPFC, right parietal‐cortex and right insula during WM conditions	NA
Alyagon et al. (2020)	Pre, post, FU‐1M	Clinical assessment: CAARS, BRIEF‐A, BAARS, BDI Behavioral tasks: mind streams, the stop signal task	The real rPFC TMS stimulation can induce alleviation of adults' ADHD symptoms, compared to AC and sham stimulation. The response rates were relatively modest	AC group: seizure, transient headaches and scalp discomfort localized to the stimulation area
Cao et al. (2019)	Pre, post	Clinical assessment: SNAP‐IV Detection of serum miRNAs	The rTMS treatment or ATX administration showed significant improvement in AD, HI and OD. the sham rTMS or placebo sham rTMS or placebo failed to cause any obvious improvements	Real rTMS group: One patient reported headache
Cao et al. (2018)	Pre, post	Clinical assessment: SNAP‐IV Executive function test: CPT, WISC, IGT	The scores of all factors in the SNAP‐IV questionnaire were lower than those before treatment in the three groups; the scores of three subtests of WISC, CPT, and IGT were also significantly higher than those before treatment. The rTMS + ATX group had a better improvement in AD and HI on the SNAP‐IV questionnaire compared with the other groups and also had a higher efficacy on cold and hot executive functions such as arithmetic, forward numbers, coding, and IGT. In addition, the ATX group performed better than the rTMS group in coding and IGT	Mild scalp discomfort and headache
Paz et al. (2018)	Pre, mid, post, FU‐1W	CAARS, TOVA	No differences in clinical outcomes were detected between the actual dTMS and sham groups	Headache
Weaver et al. (2012)	Pre, mid, post	CGI, ADHD‐IV scales	There was an overall significant improvement in the clinical global impression of improvement and the ADHD‐IV scales across the study phases	Mild headaches and scalp discomfort were reported in a minority (*n* = 3)
Bloch et al. (2010)	Pre/post	PANAS, VASs, CANTAB	There was a specific beneficial effect on attention after a real rTMS course, but it had no effect on measures of mood and anxiety. The sham rTMS had no effect	NA

Abbreviations: AAQoL, the adult ADHD quality of life measure; AC, active control; AD, attention deficit; ADHD, attention‐deficit hyperactivity disorder; ATX, atomoxetine; BAARS, Barkely Adult ADHD Rating Scale; BDI, Beck's Depression Inventory; BRIEF‐A, behavior rating inventory of executive function–adult version; CAARS, the Conners' Adult ADHD Rating Scale; CANTAB, Cambridge Neuropsychological Test Automated Battery; CGAS, children's global assessment scale; CGI, clinical global impression; CPRS‐R‐L, the Conners' Parent Rating Scale‐Revised The Long Form; CPT, continuous performance test; dTMS, deep transcranial magnetic stimulation; FU, follow up; HI, hyperactivity impulse; IGT, Iowa Gambling Task; M, month; Mid, mid‐treatment; NA, not available OD, oppositional defiance; PANAS, the positive and negative affect schedule; Post, post‐treatment; Pre, pretreatment; rDLPFC, right dorsolateral prefrontal cortices; rPFC, right prefrontal cortex; rTMS, repetitive transcranial magnetic stimulation; SNAP‐IV, the Swanson, Nolan, and Pelham, Version IV; T.O.V.A., test of variables of attention; VASs, visual analogue scales; W, week; WISC, Wechsler Intelligence Scale for Children; WM, working memory.

### 3.3 Quality Appraisal of Literatures

Risk assessment for the included parallel‐controlled trials is presented in Table 4, and risk assessment for cross‐over trials is presented in Table 5. Three articles were rated as high risk (Cao et al. 2019, 2018, 2019, 2018; Paz et al. 2018), with Paz et al.'s (2018) study rated as high risk due to receiving funding from a private company. Ratings using the GRADE methodology for all outcome measurements were inconsistent and ranged from low to very low quality (see Table S2); therefore, most studies were classified as fair.

**TABLE 4:** The Cochrane risk of bias tool for randomized controlled trials.

Article, year	Random sequence generation	Allocation concealment	Blinding of participants and personnel	Blinding of outcome assessments	Incomplete outcome data	Selective reporting	Other bias
Nagy et al. (2022)	Low	Low	Low	Low	Low	Low	Unclear
Bleich et al. (2021)	Low	Low	Low	Low	Low	Low	Unclear
Alyagon et al. (2020)	Low	Low	Low	Low	Low	Low	Unclear
Cao et al. (2019)	Low	Unclear	High	Low	Low	Low	Unclear
Cao et al. (2018)	Low	Low	High	Low	Low	Low	Unclear
Paz et al. (2018)	Unclear	Unclear	Low	Low	Low	Low	High

**TABLE 5:** The Cochrane risk of bias tool for randomized crossover trials.

Article, year	Random sequence generation	Carry‐over effect	Incomplete outcome data	Selective reporting	Blinding of participants and personnel	Blinding of outcome assessments
Weaver et al. (2012)	Low	Low	Low	Low	Low	Low
Bloch et al. (2010)	Low	Low	Low	Low	Low	Low

## 4 Meta‐Analysis

### 4.1 Inattention Symptoms

**FIGURE 2:**
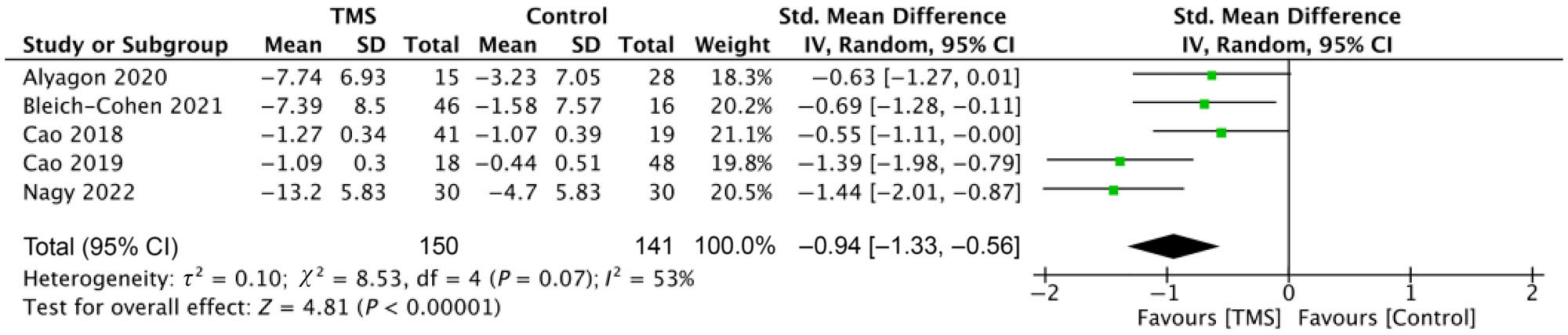
Forest plot of effect size for comparing the difference in the short‐term improvement of inattention symptoms between TMS and control groups. CI, confidence interval; Std, standardized; TMS, transcranial magnetic stimulation.

**FIGURE 3:**
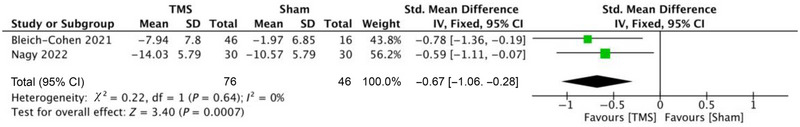
Forest plot of effect size for comparing the difference in the long‐term improvement of inattention symptoms between TMS and control groups. CI, confidence interval; Std, standardized; TMS, transcranial magnetic stimulation.

A total of five studies (Alyagon et al. 2020; Bleich‐Cohen et al. 2021; Cao et al. 2019, 2018, 2019, 2018; Nagy et al. 2022) reported immediate improvement in ADHD symptoms in five studies, involving 150 patients. Two of the studies (Bleich‐Cohen et al. 2021; Nagy et al. 2022), involving 76 patients, also reported improvements at follow‐up after 1 month. DSM‐IV, CAARS, and SNAP‐IV scale were used to assess the symptoms of inattention, and higher scores indicated more severe symptoms. Meta‐analysis and pooled analysis showed that TMS had an immediate improvement in the symptoms of inattention in ADHD patients (SMD = −0.94, 95% CI = −1.33 to −0.56, *p* < 0.001), and there was a high degree of heterogeneity (*τ* ^2^ = 0.10; *χ* ^2^ = 8.53, df = 4, *p* = 0.07; *I* ^2^ = 53%; Figure 2). And after 1 month of follow‐up, the TMS group still had significantly better symptoms of inattention than the control group (SMD = −0.67, 95% CI = −1.06 to 0.28, *p* < 0.001), with low heterogeneity (*χ* ^2^ = 0.22, df = 1, *p* = 0.64; *I* ^2^ = 0%; Figure 3).

### 4.2 Hyperactivity/Impulsivity Symptoms

**FIGURE 4:**
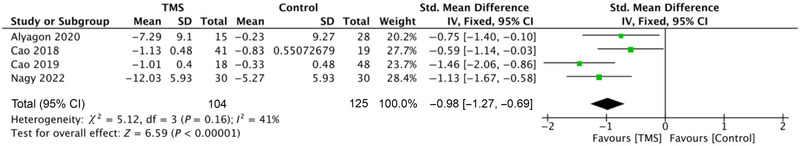
Forest plot of effect size for comparing the difference in the short‐term improvement of hyperactivity/impulsivity symptoms between TMS and control groups. CI, confidence interval; Std, standardized; TMS, transcranial magnetic stimulation.

Four studies (Alyagon et al. 2020; Cao et al. 2019, 2018, 2019, 2018; Nagy et al. 2022) evaluated the immediate effects of TMS on hyperactivity/impulsivity symptoms, involving 104 patients. DSM‐IV, CAARS, and the SNAP‐IV scale were used to evaluate the hyperactivity/impulsivity symptoms, a higher score means more severe symptoms. The synthesized results showed that TMS had an immediate improvement in hyperactivity/impulsivity symptoms in ADHD patients (SMD = −0.98, 95% CI = −1.27 to −0.69, *p* < 0.001) with low heterogeneity (*χ* ^2^ = 5.12, df = 3, *p* = 0.16; *I* ^2^ = 41%; Figure 4). As for the 1‐month follow‐up for improvement in hyperactivity/impulsivity symptoms, Nagy et al. (2022) showed that there was no significant difference between the TMS intervention and control groups. The study by Bleich‐Cohen et al. (2021) found that there was no significant interaction between the TMS intervention and control groups based on changes in CAARS subscales.

### 4.3 ADHD Total Symptoms

**FIGURE 5:**
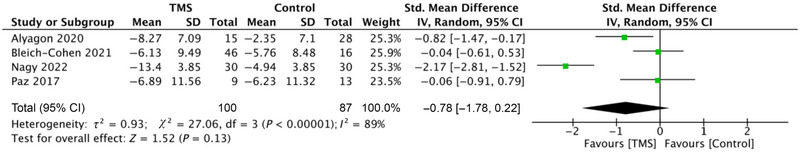
Forest plot of effect size for comparing the difference in the short‐term improvement of ADHD total symptoms between TMS and control groups. CI, confidence interval; Std, standardized; TMS, transcranial magnetic stimulation.

**FIGURE 6:**
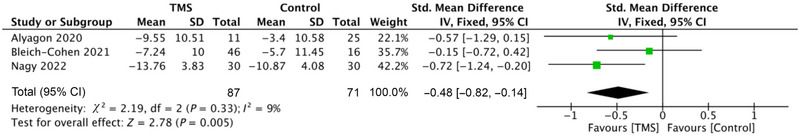
Forest plot of effect size for comparing the difference in the long‐term improvement of ADHD total symptoms between TMS and control groups. CI, confidence interval; Std, standardized; TMS, transcranial magnetic stimulation.

Four studies (Alyagon et al. 2020; Bleich‐Cohen et al. 2021; Nagy et al. 2022; Paz et al. 2018) reported the immediate effects of TMS on ADHD total symptoms, involving 100 ADHD patients. Three of the studies (Alyagon et al. 2020; Bleich‐Cohen et al. 2021; Nagy et al. 2022) also reported the results of 1‐month follow‐up. DSM‐IV and CAARS were used to evaluate the total symptoms of ADHD, with a higher score suggesting more severe symptoms. Pooled analysis showed that TMS did not immediately improve the total symptoms of ADHD patients (SMD = −0.78, 95% CI = −1.78 to 0.22, *p* = 0.13), and the heterogeneity was high (*τ* ^2^ = 0.93; *χ* ^2^ = 27.06, df = 3, *p* < 0.00001; *I* ^2^ = 89%; Figure 5). However, there was an improvement in outcomes at 1‐month follow‐up (SMD = −0.48, 95% CI = −0.82 to −0.14, *p* = 0.005), and heterogeneity was low (*χ* ^2^ = 2.19, df = 2, *p* = 0.33; *I* ^2^ = 9%; Figure 6).

### 4.4 Adverse Events

Regarding the side effects of TMS, in our included studies, no significant side effects were reported in the included studies. But five studies reported headaches (Alyagon et al. 2020; Cao et al. 2019, 2018; Paz et al. 2018; Weaver et al. 2012), three of which also reported scalp discomfort (Alyagon et al. 2020; Cao et al. 2018; Weaver et al. 2012). The remaining three studies did not report adverse events (Bleich‐Cohen et al. 2021; Bloch et al. 2010; Nagy et al. 2022). Headache was the most reported by patients After TMS intervention, followed by mild scalp discomfort. These side effects were mild and transient and the minor side effects usually resolved swiftly after the TMS intervention. Headache can be improved by reducing the intensity of the TMS (Rossi et al. 2009). Serious safety events, such as induced seizures and transient acute hypomania, were not reported in the included studies.

## 5 Discussion

This systematic review and meta‐analysis aimed to investigate the efficacy and safety of TMS in improving attention deficits, hyperactivity/impulsivity, and total symptoms in individuals with ADHD. Overall, the results of our systematic review and meta‐analysis demonstrated that ADHD patients treated with TMS had improved symptoms of inattention and hyperactivity/impulsivity after the 3–6‐week intervention compared to those treated with n ham rTMS or non‐TMS. And inattention symptoms and total ADHD symptoms improved at 1‐month follow‐up. These results were consistent with our hypothesis. The results corroborate our hypothesis that rTMS can improve ADHD symptoms, and they are also consistent with the findings from other NIBS modalities such as tDCS (Leffa et al. 2022; Salehinejad et al. 2022; Salehinejad et al. 2020). These studies further emphasize the effectiveness of NIBS techniques in the treatment of ADHD.

Compared with the control group, the TMS group had a significant improvement in inattention symptoms after intervention and 1‐month follow‐up. Studies using whole‐brain voxel‐based morphometry (VBM) and functional MRI (fMRI) showed evidence of hypoactivity in the right dorsolateral prefrontal cortex (DLPFC) in patients with ADHD during inhibitory control and attention tasks (Hart et al. 2013; Norman et al. 2016). The TMS intervention in the included studies targeted increased neural activity in attention‐related brain regions (Bleich‐Cohen et al. 2021), which may explain the improvement of inattention symptoms with TMS. Moreover, the TMS still significantly improved ADHD inattention symptoms at 1‐month follow‐up compared with non‐TMS interventions. This persistent positive effect on inattention symptoms suggested that TMS could facilitate neural plasticity. A review of studies on the role of TMS in neurological disorders also suggested that the therapeutic effects of TMS could last from a few days to a few weeks (Mosilhy et al. 2022). The physiological basis of after‐effects following rTMS remains unclear. However, there is evidence supporting the idea that these sustained effects may share similarities with LTP and LTD observed in animals (Klomjai, Katz, and Lackmy‐Vallée 2015).

Our study found an immediate improvement in hyperactivity/impulsivity symptoms in ADHD patients following TMS intervention. A preliminary study by Gómez et al. (2014) found that TMS stimulation of left DLPFC in children with ADHD can improve inattention and hyperactivity/impulsivity symptoms. Dysregulation of the frontal limbic circuit was found to be associated with symptoms of hyperactivity‐impulsivity in ADHD patients (Rubia 2018; Wasserstein and Stefanatos 2016). In the included studies, TMS extensively stimulated the DLPFC region in ADHD patients. Therefore, the significant improvement in hyperactivity/impulsivity symptoms after TMS intervention may be explained by the targeted modulation mechanism of TMS (George, Lisanby, and Sackeim 1999). However, sustained effects may require a more precise localization of the target brain region in TMS interventions for ADHD patients. This also emphasizes the importance of core symptom screening before TMS intervention to accurately identify the target brain area in the future.

Our systematic review and meta‐analysis found that TMS did not immediately improve ADHD total symptom scores, but the total scores improved in the 1‐month follow‐up. This could be explained in part by the heterogeneity of TMS intervention protocols in the included studies. Three of the studies had a short intervention duration of only 3 weeks (Alyagon et al. 2020; Bleich‐Cohen et al. 2021; Nagy et al. 2022), and it may take time for the improvement of TMS to improve ADHD total symptoms. As for improvement in total ADHD symptoms at 1‐month follow‐up, TMS treatment for neurological disorders can last from days to weeks, which is consistent with the follow‐up time in the included literature (Mosilhy et al. 2022).

A recent study shares similarities with our research (Y. Chen et al. 2023). However, our study includes two additional crossover trials, and we place greater emphasis on the improvement of core symptoms in ADHD patients through rTMS. But, the rTMS efficacy and important clinical utility for ADHD cannot be concluded, and further studies with optimized designs are needed. One of the reasons for the discrepancy in study results is the variability of stimulation parameters, which may not be suitable for all ADHD subtypes. According to the DSM‐IV classification criteria, ADHD is categorized as persistent attention deficit type (ADHD‐I), hyperactive‐impulsive dominant type (ADHD‐H), and a combination of both (ADHD‐C), depending on the clinical symptoms (Luo et al. 2021). Of the eight studies included in this systematic review and meta‐analysis, only two studies(Nagy et al. 2022; Weaver et al. 2012) differentiated between subtypes of ADHD patients included, but the effect of rTMS on different subtypes of ADHD was not reflected in the results. Other study found significant differences in cognitive functioning across ADHD subtypes, with the ADHD‐I subtype performing the worst on all cognitive domains, while the ADHD‐H subtype scored the highest and showed relatively good cognitive functioning (Molavi et al. 2020). Early studies have shown that there are neuropsychological differences in ADHD subtypes (especially ADHD‐I vs. ADHD‐C) (Nigg et al. 2002). There has also been a lot of evidence from neuroimaging studies highlighting neurobiological differences between clinical subtypes of ADHD (Saad, Griffiths, and Korgaonkar 2020). The effectiveness of neurofeedback therapy in treating children with ADHD is also subtype‐specific (Bluschke et al. 2020). So it is essential to consider the different subtypes of ADHD and analyze them in subgroups, when examining treatment outcomes.

### 5.1 Limitations and Future Directions

There were some limitations of our systematic review and meta‐analysis, which should be interpreted with caution. First, both parallel randomized controlled trials and crossover designs were included, and as the ADHD patients who are included have age differences, variability in core symptoms, distinctions in ADHD subtypes, as well as differences in whether they are receiving pharmacotherapy, the clinical heterogeneity should be noticed. Second, the follow‐up period of the analyzed studies was only 1 month in trial, this may be considered as a factor that reduces the strength of the evidence because of insufficient observation time. Third, subgroup analysis of different intervention protocols could not be conducted because of the insufficient number of included studies. Given the limitations, future research desighs could broaden thier scope, lengthen thier duration, and enhance standardization and quality. And future research on the enduring impacts of rTMS for individuals with ADHD should take into account various factors such as the patient's age, the specific subtype of ADHD, and the parameters of TMS treatment, which encompass the intensity, frequency, and the targeted brain areas for stimulation.

## 6 Conclusion

This systematic review showed that TMS significantly as well as safely improved inattention and hyperactivity/impulsivity symptoms in ADHD patients after 3–6 weeks of intervention compared to non‐TMS treatments. During 1 month of follow‐up after intervention, TMS also significantly improved the inattention symptoms and total symptom scores in ADHD patients compared to sham rTMS or non‐TMS treatments. Given the heterogeneity in the methodology and the core symptoms among ADHD patients, the interpretation of using TMS should be careful. In the future, it is necessary to clarify the association of different brain regions and symptoms in ADHD patients, which is crucial for establishing target brain areas in TMS.

## Author Contributions

**Binbin Fu**: conceptualization, data curation, formal analysis, investigation, methodology, software, visualization, writing–original draft, writing–review and editing. **Xiangyue Zhou**: conceptualization, data curation, formal analysis, investigation, methodology, software, visualization, writing–original draft, writing–review and editing. **Xuan Zhou**: conceptualization, methodology, project administration, supervision, validation, visualization, writing–review and editing. **Xin Li**: methodology, project administration, supervision, validation, visualization, writing–review and editing. **Zhengquan Chen**: formal analysis, methodology, resources, software, supervision, validation, writing–review and editing. **Yanbin Zhang**: formal analysis, methodology, resources, software, validation, writing–review and editing. **Qing Du**: conceptualization, funding acquisition, methodology, project administration, supervision, writing–review and editing.

## Conflicts of Interest

The authors declare no conflicts of interest.

7

### Peer Review

The peer review history for this article is available at https://publons.com/publon/10.1002/brb3.70246

## Supporting information



Supporting Information

## Data Availability

All data that support the findings of this study were included in this manuscript and its Supporting Information files.
