# Supplementary material for: Efficacy and Safety of Transcranial Magnetic Stimulation for Attention‐Deficit Hyperactivity Disorder: A Systematic Review and Meta‐Analysis
Source: Brain Behav. 2025 Jan 19;15(1):e70246. doi: 10.1002/brb3.70246 (PMC11743978; doi:10.1002/brb3.70246)
Supplement: Supplementary file 1 — Supporting Information [file BRB3-15-e70246-s001.docx]

**TableS1 The specific search terms**

| Source | Literature quantity | Search terms |
| --- | --- | --- |
| PubMed | 316 | (((((((Transcranial Magnetic Stimulation[MeSH Terms]) OR (Magnetics[Text Word])) OR (magnetic brain stimulation[Text Word])) OR (TMS[Text Word])) OR (rTMS[Text Word])) OR (dTMS[Text Word]))) AND ((((((((((Attention Deficit Disorder with Hyperactivity[MeSH Terms]) OR (Attention Deficit Hyperactivity Disorder[Text Word])) OR (Attention Deficit Disorder with Hyperactivity[Text Word])) OR (attention?deficit[Text Word])) OR (brain dysfunction[Text Word])) OR (hyperactiv*[Text Word])) OR (hyperkin*[Text Word])) OR (ADHD[Text Word])) OR (ADDH[Text Word])) OR (ADHS[Text Word])) |
| Web of science | 5364 | 1 (((((TS= (Transcranial Magnetic Stimulation)) OR TS=(Magnetics)) OR TS=(magnetic brain stimulation)) OR TS=(TMS)) OR TS=(rTMS)) OR TS=(dTMS) and Preprint Citation Index (Exclude – Database)  2 ((((((((TS= (Attention Deficit Disorder with Hyperactivity) OR TS=(Attention Deficit Hyperactivity Disorder)) OR TS=(Attention Deficit Disorder with Hyperactivity)) OR TS=(attention?deficit)) OR TS=(brain dysfunction)) OR TS=(hyperactiv*)) OR TS=(hyperkin*)) OR TS=(ADHD)) OR TS=(ADDH)) OR TS=(ADHS) and Preprint Citation Index (Exclude – Database)  3 #2 AND #1 and Preprint Citation Index (Exclude – Database) |
| Embase | 526 | ('transcranial magnetic stimulation': ti,ab,kw OR magnetics: ti,ab,kw OR 'magnetic brain stimulation': ti,ab,kw OR tms: ti,ab,kw OR rtms: ti,ab,kw OR dtms: ti,ab,kw) AND ('attention deficit hyperactivity disorder': ti,ab,kw OR 'attention deficit disorder with hyperactivity': ti,ab,kw OR attention?deficit: ti,ab,kw OR 'brain dysfunction': ti,ab,kw OR hyperactiv*:ti,ab,kw OR hyperkin*:ti,ab,kw OR adhd: ti,ab,kw OR addh: ti,ab,kw OR adhs: ti,ab,kw) |
| Cochrane | 643 | #1 MeSH descriptor: [Transcranial Magnetic Stimulation] explode all trees  #2 (Magnetics): ti,ab,kw OR (magnetic brain stimulation):ti,ab,kw OR (TMS):ti,ab,kw OR (rTMS):ti,ab,kw OR (dTMS):ti,ab,kw  #3 #1 OR #2  #4 MeSH descriptor: [Attention Deficit Disorder with Hyperactivity] explode all trees  #5 (Attention Deficit Hyperactivity Disorder): ti,ab,kw OR (Attention Deficit Disorder with Hyperactivity):ti,ab,kw OR (attention?deficit):ti,ab,kw OR (brain dysfunction):ti,ab,kw OR (hyperactiv*):ti,ab,kw  #6 (hyperkin*): ti,ab,kw OR (ADHD):ti,ab,kw OR (ADDH):ti,ab,kw OR (ADHS):ti,ab,kw  #7 #4 OR #5 OR #6  #8 #3 AND #7 |

**Table S2 The quality of the evidence**

Patient or population: patients with ADHD
Intervention: TMS

| Certainty assessment | | | | | | | № of patients | | Effect | | Certainty | Importance |
| --- | --- | --- | --- | --- | --- | --- | --- | --- | --- | --- | --- | --- |
| № of studies | Study design | Risk of bias | Inconsistency | Indirectness | Imprecision | Other considerations | TMS | Non-TMS | Relative (95% CI) | Absolute (95% CI) |  |  |
| **inattention symptoms** | | | | | | | | | | | | |
| 5 | randomised trials | serious^a^ | serious^b^ | not serious | serious^c^ | none | 150 | 141 | - | SMD 0.94 lower (1.33 lower to 0.56 lower) | ⨁◯◯◯ Very low | IMPORTANT |
| **inattention symptoms follow up 1 month** | | | | | | | | | | | | |
| 2 | randomised trials | not serious | not serious | not serious | extremely serious^c,d^ | none | 76 | 46 | - | SMD 0.67 lower (1.06 lower to 0.28 lower) | ⨁◯◯◯ Very low | IMPORTANT |
| **hvperactive/impulsive sympotoms** | | | | | | | | | | | | |
| 4 | randomised trials | serious^a^ | not serious | not serious | serious^c^ | none | 104 | 125 | - | SMD 0.98 lower (1.27 lower to 0.69 lower) | ⨁⨁◯◯ Low | IMPORTANT |
| **ADHD total symptoms** | | | | | | | | | | | | |
| 4 | randomised trials | not serious | serious^b^ | not serious | serious^c^ | none | 100 | 87 | - | SMD 0.78 lower (1.78 lower to 0.22 higher) | ⨁⨁◯◯ Low | IMPORTANT |
| **ADHD total symptoms follow up 1 month** | | | | | | | | | | | | |
| 3 | randomised trials | not serious | not serious | not serious | extremely serious^c,d^ | none | 87 | 71 | - | SMD 0.48 lower (0.82 lower to 0.14 lower) | ⨁◯◯◯ Very low | IMPORTANT |

CI**:** confidence interval; SMD: standardised mean difference.

#### Explanations

a. >25% of the participants was from studies with high risk of bias.

b. I^2^ > 50%.

c. Total population size is less than 300.

d. The number of studies included is small.

**Title:** Efficacy of transcranial magnetic stimulation for attention-deficit hyperactivity disorder: a systematic review and meta-analysis

**Journal name:** [*Neuropsychology Review*](https://link.springer.com/journal/11065?IFA)

**Author name:**

Binbin Fu ,^1,3 †^ (First author), B.M.

Xiangyue Zhou ^1†^ (Co-first author), B.M.

Xuan Zhou ^1^ (Co-author), M.M.

Xin Li ^1^ (Co-author), M.S.

Zhengquan Chen ,^2^ (Co-author), M.S.

Yanbin Zhang ,^2^ (Co-author), B.S.

Qing Du^1,3*^ (Corresponding author), Ph.D.

^†^ These authors contributed equally to this work.

^*^ Corresponding author.

**Affiliations**

1Department of Rehabilitation, Xinhua Hospital, Shanghai Jiao Tong University School of Medicine, Shanghai 200092, China

2Institute of Rehabilitation Engineering and Technology, University of Shanghai for Science and Technology, Shanghai 200093, China

3Chongming Branch of Xinhua Hospital, School of Medicine, Shanghai Jiao Tong University, Shanghai 202150, China

**Corresponding author**

***1. Name:*** Qing Du (Corresponding author)

***Address:*** 1665 Kongjiang Road, Shanghai 200092, China

***Tel:*** 86-021-25078600***; Fax:*** 008602165030840***;***

***Email:*** [duqing@xinhuamed.com.cn](mailto:duqing@xinhuamed.com.cn)
